# Temporal Changes in Coronary ^18^F-Fluoride Plaque Uptake in Patients with Coronary Atherosclerosis

**DOI:** 10.2967/jnumed.122.264331

**Published:** 2023-09

**Authors:** Marwa Daghem, Philip D. Adamson, Kang-Ling Wang, Mhairi Doris, Rong Bing, Edwin J.R. van Beek, Laura Forsyth, Michelle C. Williams, Evangelos Tzolos, Damini Dey, Piotr J. Slomka, Marc R. Dweck, David E. Newby, Alastair J. Moss

**Affiliations:** 1 British Heart Foundation Centre for Cardiovascular Science, University of Edinburgh, Edinburgh, United Kingdom;; 2 Christchurch Heart Institute, University of Otago, Christchurch, New Zealand;; 3 Edinburgh Imaging, Queen’s Medical Research Institute, University of Edinburgh, Edinburgh, United Kingdom;; 4 Edinburgh Clinical Trials Unit, University of Edinburgh, Edinburgh, United Kingdom;; 5 Biomedical Imaging Research Institute, Cedars–Sinai Medical Centre, Los Angeles, California; and; 6 Department of Cardiovascular Science, University of Leicester and National Institute for Health Research Leicester Biomedical Research Centre, Leicester, United Kingdom

**Keywords:** atherosclerosis, coronary calcification, PET, coronary microcalcification activity, myocardial infarction

## Abstract

Coronary ^18^F-sodium fluoride (^18^F-fluoride) uptake is a marker of both atherosclerotic disease activity and disease progression. It is currently unknown whether there are rapid temporal changes in coronary ^18^F-fluoride uptake and whether these are more marked in those with clinically unstable coronary artery disease. This study aimed to determine the natural history of coronary ^18^F-fluoride uptake over 12 mo in patients with either advanced chronic coronary artery disease or a recent myocardial infarction. **Methods:** Patients with established multivessel coronary artery disease and either chronic disease or a recent acute myocardial infarction underwent coronary ^18^F-fluoride PET and CT angiography, which was repeated at 3, 6, or 12 mo. Coronary ^18^F-fluoride uptake was assessed in each vessel by measuring the coronary microcalcification activity (CMA). Coronary calcification was quantified by measuring calcium score, mass, and volume. **Results:** Fifty-nine patients had chronic coronary artery disease (median age, 68 y; 93% male), and 52 patients had a recent myocardial infarction (median age, 65 y; 83% male). Reflecting the greater burden of coronary artery disease, baseline CMA values were higher in those with chronic coronary artery disease. Coronary ^18^F-fluoride uptake (CMA > 0) was associated with higher baseline calcium scores (294 Agatston units [AU] [interquartile range, 116–483 AU] vs. 72 AU [interquartile range, 8–222 AU]; *P* < 0.001) and more rapid progression of coronary calcification scores (39 AU [interquartile range, 10–82 AU] vs. 12 AU [interquartile range, 1–36 AU]; *P* < 0.001) than was the absence of uptake (CMA = 0). Coronary ^18^F-fluoride uptake did not markedly alter over the course of 3, 6, or 12 mo in patients with either chronic coronary artery disease or a recent myocardial infarction. **Conclusion:** Coronary ^18^F-fluoride uptake is associated with the severity and progression of coronary artery disease but does not undergo a rapid dynamic change in patients with chronic or unstable coronary artery disease. This finding suggests that coronary ^18^F-fluoride uptake is a temporally stable marker of established and progressive disease.

## Visual Abstract

**Figure:**
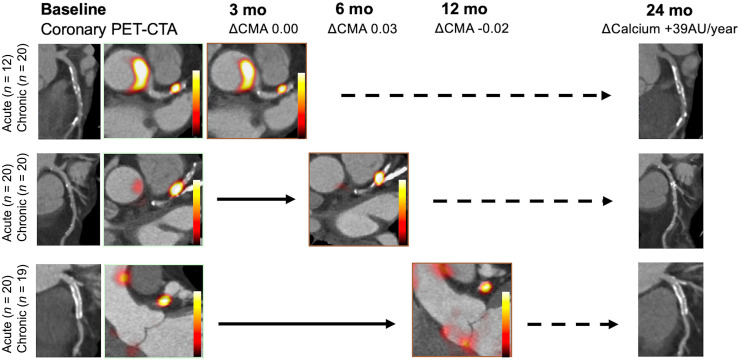

Coronary atherosclerosis is a chronic inflammatory disease that manifests as expansive plaque formation within the intima of the arterial wall and can lead to plaque rupture, coronary thrombosis, and acute myocardial infarction. In response to the atherosclerotic inflammatory cascade, small deposits of microscopic calcification accumulate in the tunica intima and represent markers of plaque activity (1). Coronary ^18^F-sodium fluoride (^18^F-fluoride) PET is a promising noninvasive imaging modality that can detect these focal regions of developing microcalcification in vivo, identifying patients at risk of future coronary atherothrombotic events (2*,*^3^). This imaging technique provides a reproducible metric of coronary microcalcification activity (CMA) (4*,*^5^) and can be used to monitor the progression of coronary artery calcium in patients with advanced coronary atherosclerosis (6).

Coronary ^18^F-fluoride uptake is observed within the territory of the culprit plaque after an acute myocardial infarction (3). Ex vivo histologic validation of coronary artery specimens has confirmed that ^18^F-fluoride colocalizes with microcalcification in the tunica intima (7). Moreover, in patients with stable chronic disease, there is increased coronary ^18^F-fluoride uptake in atherosclerotic plaques with morphologically high-risk features on intravascular ultrasound and CT, including positive remodeling and low-attenuation plaque (3). However, temporal changes in the development, evolution, and passivation of features of high-risk coronary plaque in patients at increased risk of cardiovascular events remain poorly characterized. In particular, the natural history of ^18^F-fluoride uptake within the coronary arteries of patients who have had a recent myocardial infarction is unknown. We hypothesized that coronary ^18^F-fluoride uptake would decrease over 1 y in patients with a recent type 1 myocardial infarction but not in those with stable chronic disease. To address this issue, we performed a prospective observational cohort study using serial ^18^F-fluoride PET at 3 time points over 1 y in patients with multivessel coronary artery disease and either a recent type 1 myocardial infarction or chronic stable disease.

## MATERIALS AND METHODS

### Study Design

This was an investigator-initiated prespecified prospective observational cohort study nested within 2 clinical trials of investigational medicinal products: the Prediction of Recurrent Events with ^18^F-Fluoride to Identify Ruptured and High-Risk Coronary Artery Plaques in Patients with Myocardial Infarction (PRE^18^FFIR, NCT02278211) and the Dual Antiplatelet Therapy to Inhibit Coronary Atherosclerosis and Myocardial Injury in Patients with Necrotic High-Risk Coronary Plaque Disease (DIAMOND, NCT02110303) trials (8). The nested cohort studies were approved by the local institutional review board, the Scottish Research Ethics Committee (REC references 14/SS/0089 and 16/SS/0025), the United Kingdom Administration of Radiation Substances Advisory Committee, and the Medicines and Healthcare Products Regulatory Agency. It was performed in accordance with the Declaration of Helsinki. All patients provided written informed consent before undergoing any study procedures.

### Study Population

Patients were recruited between March 2015 and July 2019. Inclusion criteria for the natural history cohort studies required the presence of multivessel coronary artery disease on invasive coronary angiography, either within 21 d of an acute type 1 myocardial infarction (NCT02278211) or in the context of advanced chronic coronary artery disease (NCT02110303). Patients were excluded if they were unable to receive iodinated contrast medium, had renal impairment (estimated glomerular filtration rate ≤ 30 mL/min per 1.73 m^2^), or were female and of child-bearing potential. Full eligibility criteria are provided in Supplemental Table 1 (supplemental materials are available at http://jnm.snmjournals.org). All patients underwent a comprehensive baseline clinical assessment including evaluation of their cardiovascular risk factor profile. The REACH (Reduction of Atherothrombosis for Continued Health) and SMART (Secondary Manifestations of Arterial Disease) risk scores were calculated. Both these scores were created specifically to predict risk in patients with established coronary artery disease (9*,*^10^).

### Image Acquisition

**FIGURE 1.**
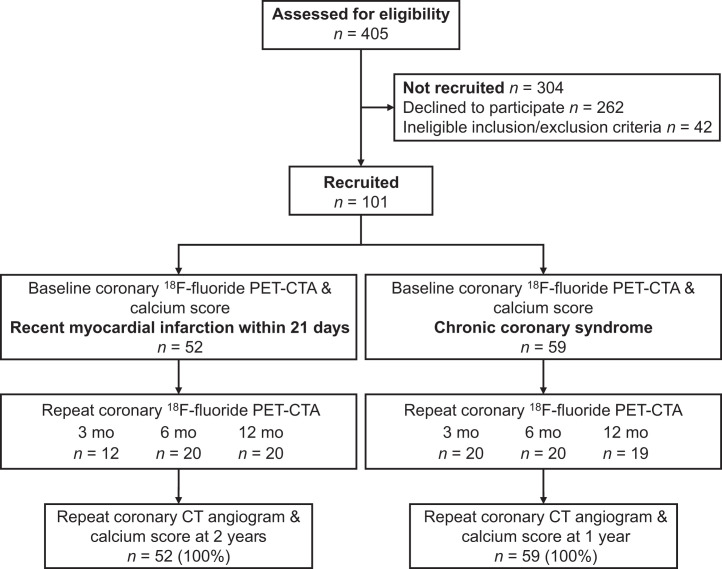
CONSORT (Consolidated Standards of Reporting Trials) diagram. CTA = CT angiography.

All patients underwent baseline ^18^F-fluoride PET and CT on a hybrid scanner (128-multidetector Biograph mCT; Siemens Medical Systems), along with unenhanced CT for calcium scoring and contrast-enhanced coronary CT angiography using a previously described standardized study protocol (4). In brief, participants with a resting heart rate of more than 65 beats/min were administered oral β-blockade (50–100 mg of metoprolol) unless contraindicated. All participants were administered a target dose of 250 MBq of intravenous ^18^F-fluoride and rested in a quiet environment. Sixty minutes after the injection, the PET acquisition was performed. Attenuation correction CT scans were performed before the acquisition of electrocardiogram-gated list-mode PET data using a single 30-min bed position centered on the heart. An electrocardiogram-gated breath-hold unenhanced CT scan (tube voltage, 120 kV; tube current based on body habitus) was performed for coronary CT calcium scoring and reconstructed in the axial plane with a 3-mm slice width and 1.5-mm increments. Finally, electrocardiogram-gated coronary CT angiography (tube voltage, 120 kV; tube current based on body habitus) was performed in mid diastole during held expiration after administration of sublingual glyceryl trinitrate. Serial contrast-enhanced coronary CT angiography and ^18^F-fluoride PET and CT were performed using the same standardized imaging protocol and on the same scanner at an interval of 3, 6, or 12 mo after the baseline scan. Unenhanced CT for calcium scoring was conducted at 12 mo (for the subgroup nested in the DIAMOND study) and 24 mo (for the subgroup nested in the PRE^18^FFIR study) (Fig. 1). To minimize exposure to ionizing radiation, all patients underwent a total of only 2 ^18^F-fluoride PET scans.

### Image Analysis

#### PET Analysis and Quantification

Electrocardiogram-gated PET images were reconstructed in diastole (50%–75% of the R–R interval, 2 iterations, 21 subsets, Siemens Ultra-HD algorithm) and fused with the contrast-enhanced coronary CT angiography images. Qualitative and semiquantitative analyses were performed independently by trained observers using a dedicated software package (FusionQuant; Cedars–Sinai Medical Centre).

CMA was used to quantify ^18^F-fluoride uptake across the coronary vasculature as described previously (2). In brief, the proximal and distal sections of the vessel (>2 mm) were identified, and a vessel-tracking algorithm was applied to extract whole-vessel tubular 3-dimensional volumes of interest from the coronary CT angiogram using dedicated semiautomated Autoplaque software (version 2; Cedars–Sinai Medical Center). These encompass all the main epicardial coronary vessels and their immediate surroundings (4-mm radius) and were used to measure the CMA (11).

Coronary ^18^F-fluoride uptake was assessed along the entire course of the coronary arteries regardless of the presence of coronary stents, and the left main stem was included in the volume of interest for the left anterior descending artery. To avoid an overspill of aortic root activity, coronary uptake at the orifice of the left main stem was excluded. CMA was defined as the average SUV within the activity volume above a background threshold defined as SUV_mean_ plus 2 SDs measured in the right atrial blood pool as described previously (2). A CMA of 0 indicated no activity, and a CMA of more than 1.56 was indicative of high activity as described previously (2).

#### Coronary Artery Calcium Score

Coronary artery calcium was quantified on a per-vessel level by an experienced observer using dedicated software (Vitrea Advanced; Toshiba Systems). Calcification was quantified as calcium score (Agatston units [AU]), calcium volume (mm^3^), and calcium mass (mg). Calcium score was derived using the Agatston method. To calculate calcium mass, a calibration factor was derived using a phantom to calculate equivalent water diameter, adjusted for body mass index and lateral diameter, and applied at a specified x-ray tube voltage (Supplemental Table 2). Because of metal artifacts, only vessels without coronary stenting were selected as part of the comparative analysis.

#### Coronary CT Angiography

The CT images were analyzed using dedicated software (Vitrea Advanced; Canon Medical Systems), with multiplanar reformatting for plaque analysis applied as necessary. Coronary arteries with diameters of at least 2 mm were assessed according to the 18-segment Society of Cardiovascular CT model (12). Disease severity was evaluated using the Duke Coronary Artery Disease Index (13), with 50% or more stenosis classified as clinically significant. The number of vessels involved, and the location of obstructive lesions (left main and proximal left anterior descending coronary arteries), were weighted according to the Duke Coronary Artery Disease Index criteria (Supplemental Table 3).

### Statistical Analysis

Continuous variables are presented as mean ± SD or as median and interquartile range as appropriate. Change in CMA was defined as the geometric mean difference in the CMA value between the baseline and the follow-up PET/CT scans after logarithmic transformation of the dataset. The Shapiro–Wilk test was used to assess normality for continuous data. Two-sample *t* testing or Wilcoxon rank-sum testing was applied to compare groups for continuous variables; the Pearson, χ^2^, or Fisher exact test was used to compare groups for categoric variables as appropriate. Pearson or Spearman rank correlation was used to assess correlations between continuous variables. All statistical analysis was performed on a per-vessel level. The statistical analyses were performed using R, version 4.0.3 (The R Foundation for Statistical Computing). A 2-sided *P* value of less than 0.05 was considered statistically significant.

## RESULTS

### Study Population

In total, 111 patients (age, 65.7 ± 7.49 y; 88.3% male) were enrolled in the prospective observational study from 2 cohorts (52 with recent acute myocardial infarction, 59 with advanced chronic coronary artery disease). All patients underwent baseline coronary ^18^F-fluoride PET, coronary CT angiography, and coronary artery calcium score imaging followed by repeat ^18^F-fluoride PET and coronary CT angiography at 3 mo (*n* = 32), 6 mo (*n* = 40), or 12 mo (*n* = 39) (Fig. 1).

Baseline demographics for both cohorts, including age, sex, traditional cardiovascular risk factors, and history of cerebrovascular disease, are demonstrated in Table 1. Patients with advanced chronic coronary artery disease had a higher Duke score (≥4 in 84% vs. 23% in those with recent myocardial infarction, *P* < 0.001). Patients with advanced chronic coronary artery disease also had higher cardiovascular risk prediction scores (REACH score of 14.0 [interquartile range, 11.5–15.5] vs. 9.0 [interquartile range, 8.0–10.0] in those with recent myocardial infarction, *P* < 0.001). All participants with advanced chronic coronary artery disease had previously undergone coronary revascularization: 25% had previous coronary artery bypass graft surgery, and 75% had a previous percutaneous coronary intervention. None of the patients with a recent myocardial infarction had prior bypass surgery, and 11% had a prior percutaneous coronary intervention, although 96% of patients underwent coronary revascularization after their index event.

**TABLE 1.** Baseline Demographics

Demographic	All participants (*n* = 111)	Advanced chronic coronary artery disease (*n* = 59)	Acute myocardial infarction (*n* = 52)	*P*
Age (y)	65.7 ± 7.49	66.7 ± 7.6	64.5 ± 7.3	NS
Sex (male)	98 (88%)	55 (93%)	43 (83%)	NS
Past medical history				
Prior acute coronary syndrome	49 (44%)	42 (71%)	45 (17%)	<0.001
Previous PCI	52 (47%)	46 (78%)	6 (12%)	<0.001
Previous CABG	15 (14%)	15 (26%)	0 (0%)	<0.001
Prior cerebrovascular disease	7 (6%)	2 (3%)	5 (10%)	
Duke score				<0.001
2	19 (18%)	1 (2%)	18 (35%)	
3	30 (28%)	8 (15%)	22 (43%)	
4	26 (24%)	19 (35%)	7 (14%)	
5	26 (24%)	23 (42%)	3 (6%)	
6	6 (6%)	4 (7%)	2 (4%)	
Cardiovascular risk factors				
Smoking habit				<0.008
Nonsmoker	43 (44%)	19 (32%)	24 (46%)	
Current smoker	28 (29%)	11 (19%)	17 (33%)	
Former smoker	26 (27%)	29 (49%)	11 (21%)	
Diabetes mellitus				
None	92 (83%)	49 (83%)	43 (83%)	NS
Type 1	2 (2%)	0 (0%)	2 (4%)	NS
Type 2	17 (15%)	10 (17%)	7 (14%)	NS
Hypertension	51 (46%)	32 (54%)	19 (37%)	NS
Total cholesterol (mmol/L)	4.44 ± 1.31	4.29 ± 0.96	4.61 ± 1.59	
Systolic blood pressure (mm Hg)	137 ± 27	146 ± 19	128 ± 31	<0.001
Medications				
Aspirin	111 (100%)	52 (100%)	59 (100%)	NS
Statin	108 (97%)	50 (96%)	58 (98%)	NS
ACE-I/ARB	90 (81%)	45 (87%)	45 (76%)	NS
β-blocker	83 (75%)	37 (71%)	46 (78%)	NS
Baseline biochemistry				
Troponin I (ng/L)	8,595 ± 16,838	10 ± 34	17,676 ± 20,633	<0.001
Creatinine (μmol/L)	82.16 ± 16.03	80.7 ± 12.8	83.7 ± 18.9	NS
Risk scores				
REACH score	11.00 (9.00–14.00)	14.00 (11.50–15.50)	9.00 (8.00–10.00)	<0.001
SMART score	18.00 (14.00–28.00)	22.00 (15.50–32.50)	15.00 (12.00–22.00)	0.004
Radiation doses				
Total dose–length product (mGy⋅cm)	821.00 (621.00–964.00)	843.00 (637.50–1134.00)	792.50 (597.00–863.75)	0.019
Initial ^18^F-fluoride dose (MBq)	244.40 (240.25–248.17)	246.02 (241.79–248.85)	243.15 (238.57–246.72)	0.015
Serial ^18^F-fluoride dose (MBq)	240.72 (236.29–246.75)	241.00 (236.03–247.35)	240.70 (237.12–245.27)	NS

NS = not statistically significant; PCI = percutaneous coronary intervention; CABG = coronary artery bypass graft; ACE-I = angiotensin-converting enzyme inhibitor; ARB = angiotensin receptor blocker.

Qualitative data are number and percentage; continuous data are median and interquartile range or mean ± SD.

### Baseline CMA

At the patient level, 72.1% (*n* = 80, CMA > 0) of patients had increased CMA, with high activity observed in 37.8% (*n* = 42, CMA > 1.56) (Table 2). At a per-vessel level, coronary ^18^F-fluoride uptake was assessed in all 330 vessels, of which 137 (41.5%) showed increased CMA at baseline (CMA > 0). Reflecting the greater burden of disease, baseline CMA was higher in those with advanced chronic coronary artery disease than in those with a recent myocardial infarction (0.17 [interquartile range, 0.00–0.96] vs. 0.00 [interquartile range, 0.00–0.18], *P* < 0.001; Table 3).

**TABLE 2.** Per-Patient Analysis of CMA over 12 Months

		3 mo			6 mo			12 mo		
Parameter	Total	No activity	Low activity	High activity	No activity	Low activity	High activity	No activity	Low activity	High activity
All participants	111									
Baseline CMA										
No activity (CMA = 0)	31/111 (28%)	5/32 (16%)	0/32 (0%)	2/32 (6%)	8/40 (20%)	2/40 (5%)	2/40 (5%)	11/39 (28%)	1/39 (3%)	0/39 (0%)
Low activity (CMA > 0 and ≤ 1.56)	38/111 (34%)	6/32 (19%)	5/32 (16%)	1/32 (3%)	5/40 (13%)	2/40 (5%)	3/40 (8%)	3/39 (8%)	8/39 (21%)	5/39 (13%)
High activity (CMA > 1.56)	42/111 (38%)	0/32 (0%)	4/32 (13%)	9/32 (28%)	1/40 (3%)	2/40 (5%)	15/40 (38%)	3/39 (8%)	3/39 (8%)	5/39 (13%)
Recent acute myocardial infarction	52									
No activity (CMA = 0)	23/52 (44%)	3/12 (25%)	0/12 (0%)	1/12 (8%)	5/20 (25%)	2/20 (10%)	1/20 (5%)	10/20 (50%)	1/20 (10%)	0/20 (0%)
Low activity (CMA > 0 and ≤ 1.56)	13/52 (25%)	2/12 (17%)	3/12 (25%)	0/12 (0%)	1/20 (5%)	1/20 (5%)	2/20 (10%)	1/20 (5%)	0/20 (0%)	3/20 (15%)
High activity (CMA > 1.56)	16/52 (31%)	0/12 (0%)	0/12 (0)%	3/12 (25%)	1/20 (5%)	2/20 (10%)	5/20 (25%)	1/20 (5%)	2/20 (10%)	2/20 (10%)
Advanced chronic coronary artery disease	59									
No activity (CMA = 0)	8/59 (14%)	2/20 (10%)	0/20 (0%)	1/20 (5%)	3/20 (15%)	0/20 (0%)	1/20 (5%)	1/19 (5%)	0/19 (0%)	0/19 (0%)
Low activity (CMA > 0 and ≤ 1.56)	25/59 (42%)	4/20 (20%)	2/20 (10%)	1/20 (5%)	4/20 (20%)	1/20 (5%)	1/20 (5%)	2/19 (11%)	8/19 (42%)	2/19 (10.5%)
High activity (CMA > 1.56)	26/59 (44%)	0/20 (0%)	4/20 (20%)	6/20 (30%)	0/20 (0%)	0/20 (0%)	10/20 (50%)	2/19 (11%)	1/19 (5%)	3/19 (16%)

**TABLE 3.** Per-Vessel Analysis of Coronary ^18^F-Fluoride Activity over 12 Months

		3 mo		6 mo		12 mo	
Parameter	Total	Negative	Positive	Negative	Positive	Negative	Positive
All participants	330						
Baseline CMA							
Negative (CMA = 0)	193/330 (58%)	44/93 (47%)	6/93 (6%)	58/120 (48%)	9//120 (8%)	69/117 (59%)	7/117 (6%)
Positive (CMA > 0)	137/330 (42%)	13/93 (14%)	30/93 (32%)	11/120 (9%)	42/120 (35%)	13/117 (11%)	28/117 (24%)
Recent acute myocardial infarction	156						
Negative (CMA = 0)	110/156 (71%)	17/36 (47%)	6/36 (17%)	32/60 (53%)	7/60 (12%)	44/60 (73%)	4/60 (7%)
Positive (CMA > 0)	46/156 (29%)	6/36 (17%)	7/36 (19%)	4/60 (7%)	17/60 (28%)	3/60 (5%)	9/60 (15%)
Advanced chronic coronary artery disease	174						
Negative (CMA = 0)	83/174 (48%)	27/57 (47%)	0/57 (0%)	26/60 (43%)	2/60 (3%)	25/57 (44%)	3/57 (5%)
Positive (CMA > 0)	91/174 (52%)	7/57 (12%)	23/57 (40%)	7/60 (12%)	25/60 (42%)	10/57 (18%)	19/57 (33%)

### CMA over 12 Mo

In patients with absence of CMA at baseline (CMA = 0), there were no discernable changes in CMA at 3, 6, or 12 mo (Table 2). Among patients with high activity at baseline (CMA > 1.56), almost all (90.5%) still demonstrated increased CMA during follow-up (Table 2). Across the entire cohort, there were no differences between baseline CMA and CMA at 3 mo (geometric mean difference, 0.00; 95% CI, −0.66 to 0.65), 6 mo (geometric mean difference, 0.03; 95% CI, −0.55 to 0.62), or 12 mo (geometric mean difference, −0.02; 95% CI, −0.59 to 0.56). This was consistent for both chronic and unstable patient cohorts.

**FIGURE 2.**
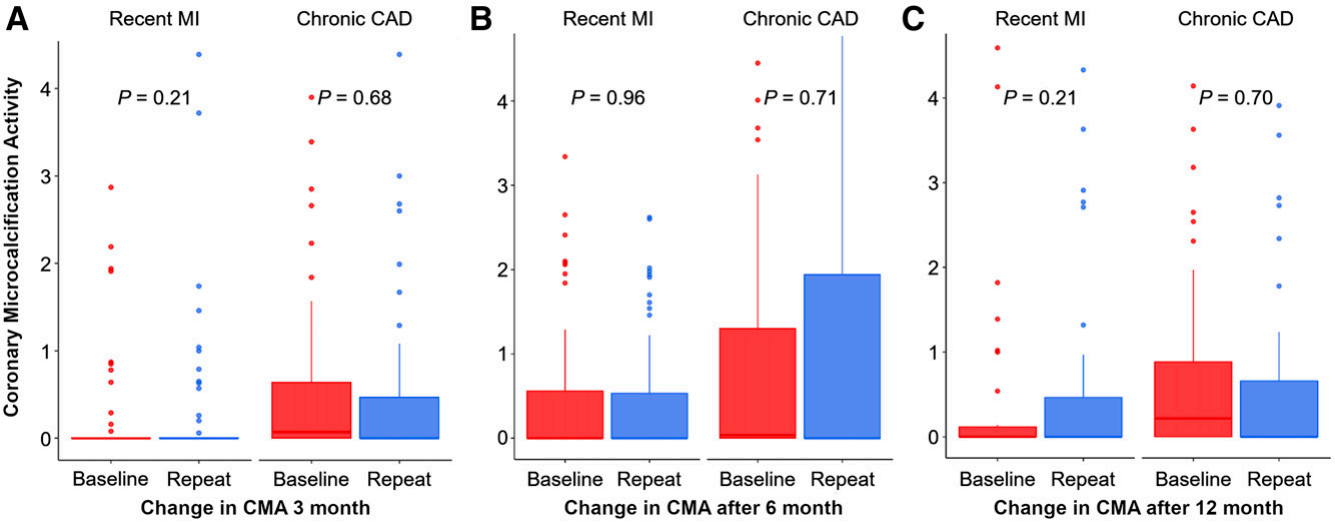
Change in ^18^F-fluoride uptake at 3, 6, and 12 mo. At vessel level, there were no significant differences in CMA uptake after 3 mo (median CMA, 0.00 [interquartile range, 0.00–0.87] vs. 0.00 [interquartile range, 0.00–0.66]; *P* = 0.79), 6 mo (median CMA, 0.00 [interquartile range, 0.00–1.11] vs. 0.00 [interquartile range, 0.00–1.40]; *P* = 0.99), or 12 mo (median CMA, 0.00 [interquartile range, 0.00–0.46] vs. 0.00 [interquartile range, 0.00–0.25]; *P* = 0.34). This was consistent for both chronic coronary artery disease and recent myocardial infarction. CAD = coronary artery disease; MI = myocardial infarction.

Similarly, at a per-vessel level, there were no differences between baseline CMA and CMA at 3, 6, or 12 mo (Fig. 2). Among vessels with no activity at baseline (CMA = 0; *n* = 193), only 3.1% (*n* = 6) had increased microcalcification activity at 3 mo, 4.7% (*n* = 9) at 6 mo, and 3.6% (*n* = 7) at 12 mo. In contrast, in vessels with activity at baseline (CMA > 0; *n* = 137 [whole cohort]), 9.5% (*n* = 13) had no activity at 3 mo, 8.0% (*n* = 11) at 6 mo, and 9.5% (*n* = 13) at 12 mo (Table 3). This was consistent for both chronic and unstable patient cohorts.

### Coronary Artery Calcification

**FIGURE 3.**
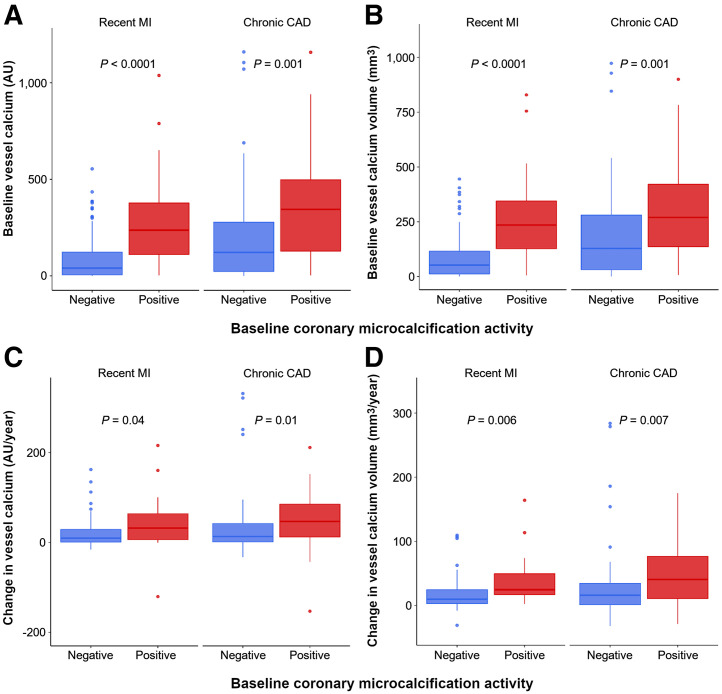
Change in coronary artery calcium score and coronary ^18^F-fluoride uptake. Vessels with increased CMA at baseline demonstrated higher baseline calcium scores (A) and calcium volume (B) and more rapid progression of calcium scores (C) and calcium volume (D) than vessels without increased ^18^F-fluoride uptake. This was consistent for both chronic coronary artery disease and recent myocardial infarction. CAD = coronary artery disease; MI = myocardial infarction.

Coronary artery calcium was assessed in all nonstented vessels at baseline. In patients with advanced chronic coronary artery disease, vessels were more calcified, with a higher baseline calcium score, higher calcium volume, and higher calcium mass, than in the patients with recent myocardial infarction (Supplemental Table 4). Overall, vessels with increased CMA had a higher baseline calcium score (294 AU [interquartile range, 116–483 AU] vs. 72 AU [interquartile range, 8–222 AU]; *P* < 0.001), higher calcium volume (268.50 mm^3^ [interquartile range, 124.50–420 mm^3^] vs. 71.50 mm^3^ [interquartile range, 15.75–219 mm^3^]; *P* < 0.001), and higher calcium mass (53.47 mg [interquartile range, 20.16–100.04 mg] vs. 13.30 mg [interquartile range, 2.07–41.49 mg]; *P* < 0.001) than vessels without increased ^18^F-fluoride uptake (Fig. 3; Supplemental Table 5). Similarly, vessels with increased CMA demonstrated more rapid progression of calcium score (39 AU [interquartile range, 10–82 AU] vs. 12 AU [interquartile range, 1–36 AU]/y; *P* < 0.001), calcium volume (32.75 mm^3^/y [interquartile range, 7.88–69 mm^3^/y] vs. 12.00 mm^3^/y [interquartile range, 1–31 mm^3^/y]; *P* = 0.001), and calcium mass (9.20 mg/y [interquartile range, 3.10–16.99 mg/y] vs. 2.60 mg/y [interquartile range, 0.39–7.36 mg/y]; *P* < 0.001) than vessels without increased CMA (Table 4; Fig. 3).

**TABLE 4.** Progression of Calcification Based on Coronary ^18^F-Fluoride Uptake at Baseline

Parameter	^18^F-fluoride uptake	No ^18^F-fluoride uptake	*P*
Number of vessels	72	120	
Change in coronary artery calcium (AU/y)	39 (10–82)	12 (1–36)	<0.001
Change in coronary artery calcium volume (mm^3^/y)	32.75 (7.88–69)	12.00 (1–31)	0.001
Change in coronary artery calcium mass (mg/y)	9.20 (3.10–16.99)	2.60 (0.39–7.36)	<0.001

Data are median and interquartile range.

A regression model to assess change in calcium volume was performed adjusting for baseline calcium volume and demonstrated no significant independent association with coronary microcalcification (β = coefficient −5.5, *P* = 0.421) (Supplemental Table 6). A regression model to assess change in calcium score (AU) was performed adjusting for baseline calcium score and demonstrated no significant independent association with coronary microcalcification (β = coefficient 4.13, *P* = 0.426) (Supplemental Table 6).

## DISCUSSION

In this prospective observational cohort study of patients with advanced chronic coronary artery disease or acute myocardial infarction, we showed that, using coronary ^18^F-fluoride, increased CMA is detectable in 3 of 4 patients and that microcalcification activity remains elevated for up to 12 mo after initial assessment. Coronary ^18^F-fluoride uptake correlates with disease burden, in terms of both coronary calcification and baseline coronary artery disease severity. Furthermore, coronary ^18^F-fluoride uptake correlated with progression of coronary artery calcification at follow-up. This correlation was consistent across a range of measures of calcification and for patients with either stable or unstable coronary artery disease. Despite these associations, we demonstrated no marked changes in coronary ^18^F-fluoride uptake over 12 mo of follow-up in either population. This finding suggests that although coronary ^18^F-fluoride uptake is a marker of disease activity, it does not change rapidly with time, consistent with the slowly evolving nature of coronary atherosclerosis.

Calcification plays an important role in the pathogenesis of atherosclerosis and begins early in the disease process (14). CT calcium scoring quantifies macroscopic deposits of calcification and provides a surrogate of total coronary atherosclerotic burden. The relationship between the coronary artery calcium score and major adverse cardiovascular events, including all-cause mortality, cardiovascular events, and nonfatal myocardial infarction, has been well established (15*,*^16^). This strong relationship occurs even though heavily calcified plaques are themselves less likely to rupture or precipitate acute myocardial infarction, the rationale being that the more plaque a patient has, the more likely it is that a clinically relevant plaque rupture will occur. Coronary artery calcification is thus a surrogate for the overall burden of coronary artery disease, which will include noncalcified high-risk plaque elsewhere in the coronary circulation.

We have here shown that both coronary ^18^F-fluoride uptake and CMA correlate with disease burden as demonstrated by the coronary artery calcium score and the Duke score. This is consistent with previous studies showing that coronary ^18^F-fluoride uptake is associated with both luminal stenosis and coronary calcification (6). We have gone on to demonstrate that coronary ^18^F-fluoride uptake predicts disease progression, with increasing uptake correlating with more rapid coronary artery calcium progression. This finding is consistent with prior studies (6*,*^17^), as well as those reporting that ^18^F-fluoride preferentially binds to pathologic mineralization and identifies areas of microcalcification (1). Indeed, ^18^F-fluoride binds more readily to regions of developing calcium and acts as a marker of calcification activity, adding distinct information to calcium scoring, which cannot differentiate between quiescent and active disease. This is supported by prior histologic data showing preferential binding of ^18^F-fluoride to developing hydroxyapatite (7).

Recent data suggest that ^18^F-fluoride PET is a potentially valuable tool in cardiovascular risk stratification. CMA represents a summary measure of ^18^F-fluoride uptake within the entire coronary vasculature and, like coronary artery calcium score, is a predictor of future myocardial infarction (5). Indeed, coronary ^18^F-fluoride uptake and CMA are associated with an increased risk of future myocardial infarction independent of age, sex, cardiovascular risk factors, segment involvement, coronary artery calcium score, coronary stents, coronary stenosis, Duke score, and recent myocardial infarction (5). These future myocardial infarction events occur over many years, and we wished to assess the time course of coronary ^18^F-fluoride uptake and CMA to determine its temporal stability as a measure of coronary artery disease activity. We therefore assessed these measures over differing timelines over a 1-y period in patients with stable and unstable coronary artery disease. We report that there is no major discernible change over a 3-, 6-, or 12-mo period irrespective of the stability of coronary artery disease. This finding suggests that biologic stabilization and healing of coronary atherosclerotic plaque are slow, that plaque activity and vulnerability may be prolonged, and that active coronary calcification persists for many months or indeed years.

Our findings do not undermine the utility of coronary ^18^F-fluoride uptake in the identification of metabolically active plaques in patients with coronary artery disease with ongoing calcifying activity and vascular inflammation. Atherosclerosis starts early in life with a long quiescent phase before the manifestation of overt disease. Before the fourth decade of life, subclinical noncalcified plaque forms in the absence of detectable coronary macrocalcification (18). Without intervention, noncalcified plaque will accumulate at approximately 1 mm^3^ per annum, and although statins can accelerate transformation to a calcified phenotype, the increased rate of calcific progression is only 1.27 mm^3^ per annum (19). Over many decades, these small differences are amplified and may in part explain the heterogeneity of coronary artery disease presentations in later life (20). This observation of slow incremental change is supported by intravascular imaging studies that reported 1%–2% volumetric changes in dense calcification over 12 mo (21). However, coronary plaques do not all follow a linear trajectory. Although most thin-capped fibroatheromas will heal over time, a smaller proportion of plaques with intensely active atherosclerosis may transform into a more vulnerable phenotype (20*,*^21^)—hence the rationale for monitoring disease activity using ligand-specific radiotracers. ^18^F-FDG has had limited clinical application in the coronary vasculature due to overspill of activity from the myocardium. In the carotid arteries, ^18^F-FDG produces a more diffuse uptake pattern along the course of the vessel, as opposed to the discrete signal of ^18^F-fluoride, which colocalizes to regions of disrupted laminar blood flow (22*,*^23^). More recently, in vivo models have suggested that ^18^F-FDG uptake does not represent merely macrophage infiltration and that this diffuse pattern of activity may be more closely aligned with medial smooth muscle uptake (24). This possibility makes it difficult to discern whether the early reduction in ^18^F-FDG signal intensity that follows the initiation of plaque-directed therapy is wholly due to a change in inflammatory cell activity in intimal plaque (25–27). Preclinical animal studies suggest that vascular inflammation and osteogenesis progress in close proximity to, and increase in parallel with, plaque progression (28*,*^29^). ^18^F-fluoride colocalizes with the distribution of osteopontin and Runx-2, established markers of early calcification activity and adverse plaque formation (7). Analogously, microcalcification is itself associated with markers of plaque vulnerability, such as intraplaque hemorrhage (30), and its presence in the fibrous cap might promote cavitation-induced plaque rupture (31). Paradoxically, macrocalcification represents the end stages of disease, with the formation of homogeneous or sheetlike calcification that effectively walls off the inflamed necrotic core and stabilizes the plaque.

Our findings are consistent with a slow time course in which active disease changes slowly before activity burns out and the plaque becomes quiescent. Microcalcification is a prolonged process compared with active acute inflammation, which is usually short-lived and changes rapidly over time. ^18^F-fluoride cannot track the early remodeling changes that have been observed with ^18^F-FDG after acute myocardial infarction (27), and our hypothesis that the coronary ^18^F-fluoride signal would decrease after acute myocardial infarction was wrong. The longer duration associated with microcalcifications may enable coronary ^18^F-fluoride uptake to detect high-risk plaques at varying phases of atheromatous progression. Moreover, such qualities do make it a more attractive risk marker for future clinical events, as is consistent with our previous finding that baseline coronary ^18^F-fluoride uptake and CMA predicted subsequent myocardial infarction at a median of 5 y of follow-up (5). Such a marker of prolonged downstream events is attractive and negates the need for short-term serial scanning or the possibility of false-positive or -negative findings if there was presence or absence of transient inflammation.

We should acknowledge several limitations of our study. Although, to our knowledge, our study included the largest number of consecutive prospectively enrolled patient cohorts to undergo repeat coronary PET and CT angiography for the dynamic assessment of coronary ^18^F-fluoride uptake, we recognize that this was a single-center study comprising a largely White male population. Because of the high level of coronary revascularization and stent implantation in our patient cohorts, quantitative analysis of coronary plaque burden was challenging to perform. Future studies exploring the relationship between quantitative plaque characteristics and burden on coronary CT angiography and coronary ^18^F-fluoride uptake on PET would be important to evaluate the added value of CMA. Finally, our patient populations all received guideline-directed medical therapy including high use of antiplatelet, statin, and renin–angiotensin system inhibitor therapies. As such, we cannot exclude the modifying effects of the treatment interventions, which are likely to be conservative, on our findings.

## CONCLUSION

Coronary ^18^F-fluoride uptake correlates with both coronary artery calcification and disease severity and is a determinant of coronary artery disease progression, irrespective of the stability of coronary artery disease. Coronary ^18^F-fluoride uptake was relatively constant over the short term, with no change in activity over 3–12 mo even in patients with recent myocardial infarction. This finding suggests that coronary ^18^F-fluoride uptake identifies established and progressive disease that can take considerable time to change and to modify.

## DISCLOSURE

This study was funded by an unrestricted educational grant from AstraZeneca. Marc Dweck, Michelle Williams, David Newby, and Alastair Moss are supported by the British Heart Foundation (FS/17/79/33226, FS/14/78/31020, CH/09/002, RE/18/5/34216, AA/18/3/34220, and FS/ICRF/20/26002). David Newby is a recipient of a Wellcome Trust Senior Investigator Award (WT103782AIA) and has received honoraria for consultancy and lectures from AstraZeneca. Edwin van Beek is supported by the Scottish Imaging Network: A Platform of Scientific Excellence (SINAPSE). The Edinburgh Clinical Research Facility and Edinburgh Imaging Facility are supported by the National Health Service Research Scotland (NRS) through the National Health Service Lothian Health Board. Michelle Williams has given lectures for Canon Medical Systems. No other potential conflict of interest relevant to this article was reported.

## Notes

KEY POINTS **QUESTION:** What is the natural history of coronary ^18^F-fluoride uptake in patients with high-risk coronary artery disease? **PERTINENT FINDINGS:** In this prospective observational cohort study, coronary ^18^F-fluoride uptake is a marker of atherosclerotic disease activity and plaque progression. Once detected, increased CMA is a temporally stable marker of risk that remains elevated for up to 12 mo. **IMPLICATIONS FOR PATIENT CARE:** The detection of CMA may allow clinicians to stratify the use of novel therapies targeted to passivating plaque activity, potentially reducing cardiovascular events in this group.

## References

[1] Irkle A Vesey AT Lewis DY. Identifying active vascular microcalcification by ^18^F-sodium fluoride positron emission tomography. Nat Commun. 2015;6:7495. 26151378 10.1038/ncomms8495 PMC4506997

[2] Kwiecinski J Tzolos E Adamson PD. Coronary ^18^F-sodium fluoride uptake predicts outcomes in patients with coronary artery disease. J Am Coll Cardiol. 2020;75:3061–3074. 32553260 10.1016/j.jacc.2020.04.046 PMC7380446

[3] Joshi NV Vesey AT Williams MC. ^18^F-fluoride positron emission tomography for identification of ruptured and high-risk coronary atherosclerotic plaques: a prospective clinical trial. Lancet. 2014;383:705–713. 24224999 10.1016/S0140-6736(13)61754-7

[4] Moss AJ Doris MK Andrews JPM. Molecular coronary plaque imaging using ^18^F-fluoride. Circ Cardiovasc Imaging. 2019;12:e008574. 31382765 10.1161/CIRCIMAGING.118.008574 PMC7668410

[5] Tzolos E Kwiecinski J Lassen ML. Observer repeatability and interscan reproducibility of ^18^F-sodium fluoride coronary microcalcification activity. J Nucl Cardiol. 2022;29:126–135. 10.1007/s12350-020-02221-1 PMC7728624 32529531

[6] Doris MK Meah MN Moss AJ. Coronary ^18^F-fluoride uptake and progression of coronary artery calcification. Circ Cardiovasc Imaging. 2020;13:e011438. 33297761 10.1161/CIRCIMAGING.120.011438 PMC7771641

[7] Moss AJ Sim AM Adamson PD. Ex vivo ^18^F-fluoride uptake and hydroxyapatite deposition in human coronary atherosclerosis. Sci Rep. 2020;10:20172. 33214599 10.1038/s41598-020-77391-6 PMC7677392

[8] Moss AJ Dweck MR Doris MK. Ticagrelor to reduce myocardial injury in patients with high-risk coronary artery plaque. JACC Cardiovasc Imaging. 2020;13:1549–1560. 31422134 10.1016/j.jcmg.2019.05.023 PMC7342015

[9] Wilson PWF D’Agostino R Bhatt DL. An international model to predict recurrent cardiovascular disease. Am J Med. 2012;125:695–703.e1. 22727237 10.1016/j.amjmed.2012.01.014

[10] Dorresteijn JAN Visseren FLJ Wassink AMJ. Development and validation of a prediction rule for recurrent vascular events based on a cohort study of patients with arterial disease: the SMART risk score. Heart. 2013;99:866–872. 23574971 10.1136/heartjnl-2013-303640

[11] Kwiecinski J Cadet S Daghem M. Whole-vessel coronary ^18^F-sodium fluoride PET for assessment of the global coronary microcalcification burden. Eur J Nucl Med Mol Imaging. 2020;47:1736–1745. 31897586 10.1007/s00259-019-04667-z PMC7271818

[12] Leipsic J Abbara S Achenbach S. SCCT guidelines for the interpretation and reporting of coronary CT angiography: a report of the Society of Cardiovascular Computed Tomography Guidelines Committee. J Cardiovasc Comput Tomogr. 2014;8:342–358. 25301040 10.1016/j.jcct.2014.07.003

[13] Mark DB Nelson CL Califf RM. Continuing evolution of therapy for coronary artery disease. Initial results from the era of coronary angioplasty. Circulation. 1994;89:2015–2025. 8181125 10.1161/01.cir.89.5.2015

[14] Pugliese G Iacobini C Fantauzzi CB Menini S. The dark and bright side of atherosclerotic calcification. Atherosclerosis. 2015;238:220–230. 25528431 10.1016/j.atherosclerosis.2014.12.011

[15] Budoff MJ Shaw LJ Liu S. Long-term prognosis associated with coronary calcification observations from a registry of 25,253 patients. J Am Coll Cardiol. 2007;49:1860–1870. 17481445 10.1016/j.jacc.2006.10.079

[16] Shaw LJ Giambrone AE Blaha MJ. Long-term prognosis after coronary artery calcification testing in asymptomatic patients: a cohort study. Ann Intern Med. 2015;163:14–21. 26148276 10.7326/M14-0612

[17] Bellinge JW Francis RJ Lee SC. ^18^F-sodium fluoride positron emission tomography activity predicts the development of new coronary artery calcifications. Arterioscler Thromb Vasc Biol. 2021;41:534–541. 33267660 10.1161/ATVBAHA.120.315364

[18] Osborne-Grinter M Kwiecinski J Doris M. Association of coronary artery calcium score with qualitatively and quantitatively assessed adverse plaque on coronary CT angiography in the SCOT-HEART trial. Eur Heart J Cardiovasc Imaging. 2022;23:1210–1221. 34529050 10.1093/ehjci/jeab135 PMC9612790

[19] Lee S-E Chang H-J Sung JM. Effects of statins on coronary atherosclerotic plaques: the PARADIGM study. JACC Cardiovasc Imaging. 2018;11:1475–1484. 29909109 10.1016/j.jcmg.2018.04.015

[20] Hwang D Kim HJ Lee S-P. Topological data analysis of coronary plaques demonstrates the natural history of coronary atherosclerosis. JACC Cardiovasc Imaging. 2021;14:1410–1421. 33454260 10.1016/j.jcmg.2020.11.009

[21] Kubo T Maehara A Mintz GS. The dynamic nature of coronary artery lesion morphology assessed by serial virtual histology intravascular ultrasound tissue characterization. J Am Coll Cardiol. 2010;55:1590–1597. 20378076 10.1016/j.jacc.2009.07.078

[22] Evans NR Tarkin JM Chowdhury MM. Dual-tracer positron-emission tomography for identification of culprit carotid plaque and pathophysiology in vivo. Circ Cardiovasc Imaging. 2020;13:e009539. 32164454 10.1161/CIRCIMAGING.119.009539

[23] Vesey AT Jenkins WSA Irkle A. ^18^F-fluoride and ^18^F-fluorodeoxyglucose positron emission tomography after transient ischemia attack or minor ischemic stroke: case-control study. Circ Cardiovasc Imaging. 2017;10:e004976. 28292859 10.1161/CIRCIMAGING.116.004976 PMC5367506

[24] Al-Mashhadi RH Tolbod LP Bloch LØ. ^18^Fluorodeoxyglucose accumulation in arterial tissues determined by PET signal analysis. J Am Coll Cardiol. 2019;74:1220–1232. 31466620 10.1016/j.jacc.2019.06.057

[25] Tawakol A Fayad ZA Mogg R. Intensification of statin therapy results in a rapid reduction in atherosclerotic inflammation: results of a multicenter fluorodeoxyglucose-positron emission tomography/computed tomography feasibility study. J Am Coll Cardiol. 2013;62:909–917. 23727083 10.1016/j.jacc.2013.04.066

[26] Elkhawad M Rudd JHF Sarov-Blat L. Effects of p38 mitogen-activated protein kinase inhibition on vascular and systemic inflammation in patients with atherosclerosis. JACC Cardiovasc Imaging. 2012;5:911–922. 22974804 10.1016/j.jcmg.2012.02.016

[27] Gaztanaga J Farkouh M Rudd JHF. A phase 2 randomized, double-blind, placebo-controlled study of the effect of VIA-2291, a 5-lipoxygenase inhibitor, on vascular inflammation in patients after an acute coronary syndrome. Atherosclerosis. 2015;240:53–60. 25752438 10.1016/j.atherosclerosis.2015.02.027

[28] Creager MD Hohl T Hutcheson JD. ^18^F-fluoride signal amplification identifies microcalcifications associated with atherosclerotic plaque instability in positron emission tomography/computed tomography images. Circ Cardiovasc Imaging. 2019;12:e007835. 30642216 10.1161/CIRCIMAGING.118.007835 PMC6338081

[29] Nadra I Mason JC Philippidis P. Proinflammatory activation of macrophages by basic calcium phosphate crystals via protein kinase C and MAP kinase pathways. Circ Res. 2005;96:1248–1256. 15905460 10.1161/01.RES.0000171451.88616.c2

[30] Lin R Chen S Liu G Xue Y Zhao X. Association between carotid atherosclerotic plaque calcification and intraplaque hemorrhage. Arterioscler Thromb Vasc Biol. 2017;37:1228–1233. 28450297 10.1161/ATVBAHA.116.308360

[31] Kelly-Arnold A Maldonado N Laudier D. Revised microcalcification hypothesis for fibrous cap rupture in human coronary arteries. Proc Natl Acad Sci USA. 2013;110:10741–10746. 23733926 10.1073/pnas.1308814110 PMC3696743

